# Predicting hidden bulk phases from surface phases in bilayered Sr_3_Ru_2_O_7_

**DOI:** 10.1038/s41598-017-10780-6

**Published:** 2017-08-31

**Authors:** Pablo Rivero, Rongying Jin, Chen Chen, Vincent Meunier, E. W. Plummer, William Shelton

**Affiliations:** 1 Center for Computation and Technology, Louisiana State University, Baton Rouge, Louisiana 70803 USA; 2 Department of Physics and Astronomy, Louisiana State University, Baton Rouge, Louisiana 70803 USA; 3 Department of Physics, Applied Physics, and Astronomy, Rensselaer Polytechnic Institute, Troy, NY 12180 USA

## Abstract

The ability to predict hidden phases under extreme conditions is not only crucial to understanding and manipulating materials but it could also lead to insight into new phenomena and novel routes to synthesize new phases. This is especially true for Ruddlesden-Popper perovskite phases that possess interesting properties ranging from superconductivity and colossal magnetoresistance to photovoltaic and catalytic activities. In particular, the physical properties of the bilayer perovskite Sr_3_Ru_2_O_7_ at the surface are intimately tied to the rotation and tilt of the RuO_6_ octahedra. To take advantage of the extra degree of freedom associated with tilting we have performed first principles hybrid density functional simulations of uniaxial pressure applied along the *c*-axis of bulk Sr_3_Ru_2_O_7_ where we find that the octahedra become tilted, leading to two phase transitions. One is a structural transition at $\simeq$1.5 GPa, and the other is from a ferromagnetic (FM) metal to an antiferromagnetic (AFM) insulator at $\simeq$21 GPa whose AFM spin configuration is different from the AFM state near the FM ground state.

## Introduction

Understanding and exploiting materials in extreme environments is crucial not only to address global energy challenges, but also to control and tailor the materials response to enhance performance, lifetime, and enable new technologies. Thus, a central goal of the materials community is to understand and control the behavior of materials that are either driven far from their equilibrium or placed in extreme conditions such as high pressure, temperature, strain or under high magnetic or electric fields. The response of a material exposed to such environments provides information on its internal structure and dynamics. To be able to assess global and local materials stability between competing structures will enable the design of new synthetic routes for stabilizing hidden metastable phases that contain highly desirable properties.

The search for hidden phases in complex materials is of central interest since it could lead to new materials that display fundamentally interesting and technologically desirable properties such as high strength, ductility or hardness and others such as colossal magnetoresistance, high temperature superconductivity or ferroelectricity. These phases may be accessed and controlled by tuning a number of parameters such as high pressure, high magnetic field, etc. The emergence of new properties is due to the intimate coupling between charge, lattice, and spin degrees of freedom. A possible approach to access new phases is the manipulation of this coupling through changes in the structural, electronic, and magnetic properties by inducing broken symmetry at the surface, interface, or local symmetries in the bulk.

Our hypothesis is that understanding the phases at the surface of a complex multi-component transition-metal compound presents a window on hidden phases in the bulk, especially under extreme conditions. Here we show for the first time that theory can explain the surface structure and electronic properties of Sr_3_Ru_2_O_7_. Our success in calculating the surface properties led us to explore the effect of applying uniaxial pressure along the *c*-axis, which lead to the emergence of new structural phases. The first transition is observed at $\simeq$1.5 GPa where octahedral tilts produce a structural transition from Bbcb to Bbmm orthorhombic symmetries. At an uniaxial pressure of $\simeq$21 GPa the second transition occurs transforming the metallic ferromagnetically (FM) ordered bulk into an AFM insulator. Although the total energy difference between the FM and AFM states as a function of octahedral rotation in the bulk is quite small, the large uniaxial pressure necessary to drive the transition indicates that a significant energy barrier exists between these two states, thereby indicating a strong lattice-spin coupling. Remarkably, the hidden AFM phase is an A-type AFM (AFM-A) where Ru atoms are ferromagnetically coupled in-plane and antiferromagnetically coupled out-of-plane in each bilayer, whereas the lowest energy AFM structure at 0 GPa (AFM-I)^1^ occurs when all Ru atoms in each bilayer are coupled ferromagnetically and coupled antiferromagnetically between bilayers.

**Figure 1:**
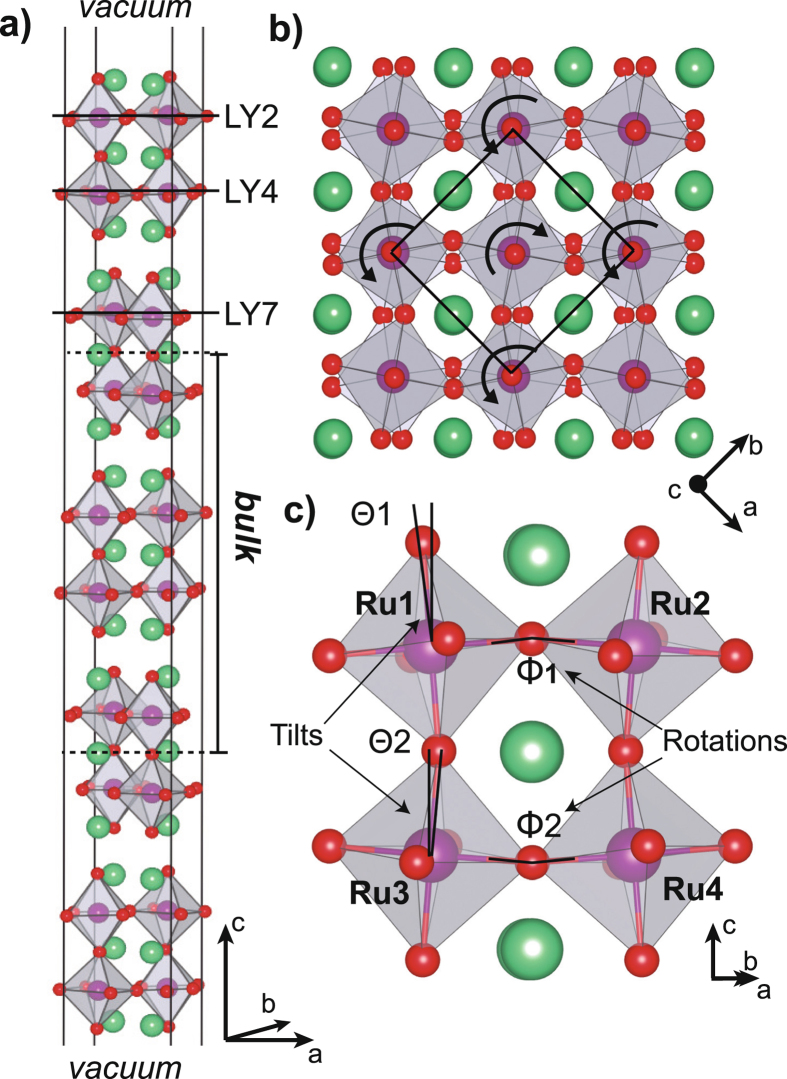
Side and top views of the orthorhombic Sr_3_Ru_2_O_7_ structure. (**a**) Model used in our calculations. Surface planes are indicated by the LYX nomenclature where X is the surface plane position. (**b**) Top view of the relaxed surface. The RuO_6_ octahedra are rotated alternately clockwise and counter-clockwise. Darker octahedra refer to the surface bilayer. (**c**) Calculated tilt and rotations observed in the surface bilayer.

Sr_3_Ru_2_O_7_ is the bilayered member of the Ruddlesden-Popper (RP) family of strontium ruthenates. The system is formed by two layers of RuO_6_ octahedra connected by an apical oxygen and interleaved by two SrO layers (Fig. 1)^2, 3^. The octahedra in the bilayer are twisted with one octahedron rotated clockwise by 8.05° about the *c* axis while the neighboring octahedra are rotated counter-clockwise by the same amount and contains no octahedral tilt. In its ground state, Sr_3_Ru_2_O_7_ is a paramagnetic (PM) metal^4, 5^ that is highly susceptible to external parameters such as pressure, composition, temperature, or magnetic field^6–8^, and defects that can produce diverse electronic and magnetic properties along with a variety of phase transitions^9–14^ including metamagnetism^15, 16^ and electron nematic phases^17^. Understanding the competing interactions in this system is key to understanding the underlying mechanisms responsible for this wide array of observed phenomena and for engineering the electronic and magnetic properties of this system.

Our experimental investigation revealed that octahedral tilt emerges when creating a (001) surface, which only occurs within the first surface bilayer^18^. Using a combination of low-energy electron diffraction (LEED) and high-resolution electron energy loss spectroscopy (HREELS) analysis, we observed more distorted RuO_6_ octahedra at the surface than in the bulk^18, 19^. These distortions are characterized by a higher rotation angle of $\simeq$10.5° ± 3° and a tilt angle of 2.6° ± 0.8° producing a surface symmetry-breaking that leads to a reduced conducting state relative to the bulk. Our computational results reproduce these structural parameters and the reduction in the conductivity. Thereby, RuO_6_ octahedral tilt is an interesting degree of freedom that can be used to investigate new phases in the bulk or at the surface.

As we will show, inducing octahedral tilt via uniaxial pressure results in a new broken symmetry that stabilizes either a new FM metallic structure or an AFM insulating phase in bulk Sr_3_ Ru_2_O_7_ depending on the pressure applied. In fact, our studies found that the charge density displays local symmetry breaking when octahedral tilt develops in the system. This symmetry breaking along with the strong FM-AFM competition in the bulk provides necessary information to postulate that the use of uniaxial pressure could trigger the FM metallic to AFM insulating transition.

## Results and Discussion

Our simulations yield two kinds of RuO_6_ octahedra tilt where the apical O atoms are pointing in opposite directions (Fig. 1). We note that while O and Sr atoms experience large displacements, the Ru atoms barely move. Our calculations yield Θ1 and Θ2 tilt angles of 1.89° and 2.00° respectively and rotation angles, Φ1 and Φ2, equal to 11° and 9.25° (see Table 1). The experimental and calculated tilts and rotation angles are in very good agreement where an enhancement of Φ1 and reduction of Φ2 relative to bulk are observed^2, 18^. The difference between Ru1-O and Ru2-O distances increased from 0 Å in the bulk to 0.033 Å at the surface indicating a symmetry breaking.

**Table 1:** Structural properties and energetics for bulk and surface (with and without RuO_6_ tilts) Sr_3_Ru_2_O_7_ systems. Θ and Φ (°) indicate tilts and rotations as described in Fig. 1c. RuX-O distances (Å) are distances between in-plane Ru and O atoms as seen in Fig. 1a. Total energy differences between FM and lowest energetically AFM phases (ΔE = E_*AFM*_ −E_*FM*_ per formula unit in meV). Stronger FM character is observed at the surface in comparison to the bulk.

	1st RuO_6_ layer				2nd RuO_6_ layer				ΔE
	Θ1	Φ1	Ru1-O	Ru2-O	Θ2	Φ2	Ru3-O	Ru4-O	
Bulk^1^	0.00	9.71	1.969	1.969	0.00	9.71	1.969	1.969	0.55
EXP-bulk^2^	0.00	8.05	1.956	1.956	0.00	8.05	1.956	1.956	
Non-tilted	0.00	10.98	1.981	1.980	0.00	9.65	1.973	1.973	11.0
Tilted	1.89	11.03	1.966	1.999	2.00	9.10	1.968	1.971	22.0
EXP-surf^18^	2.6 ± 0.8	10.5 ± 3							

**Figure 2:**
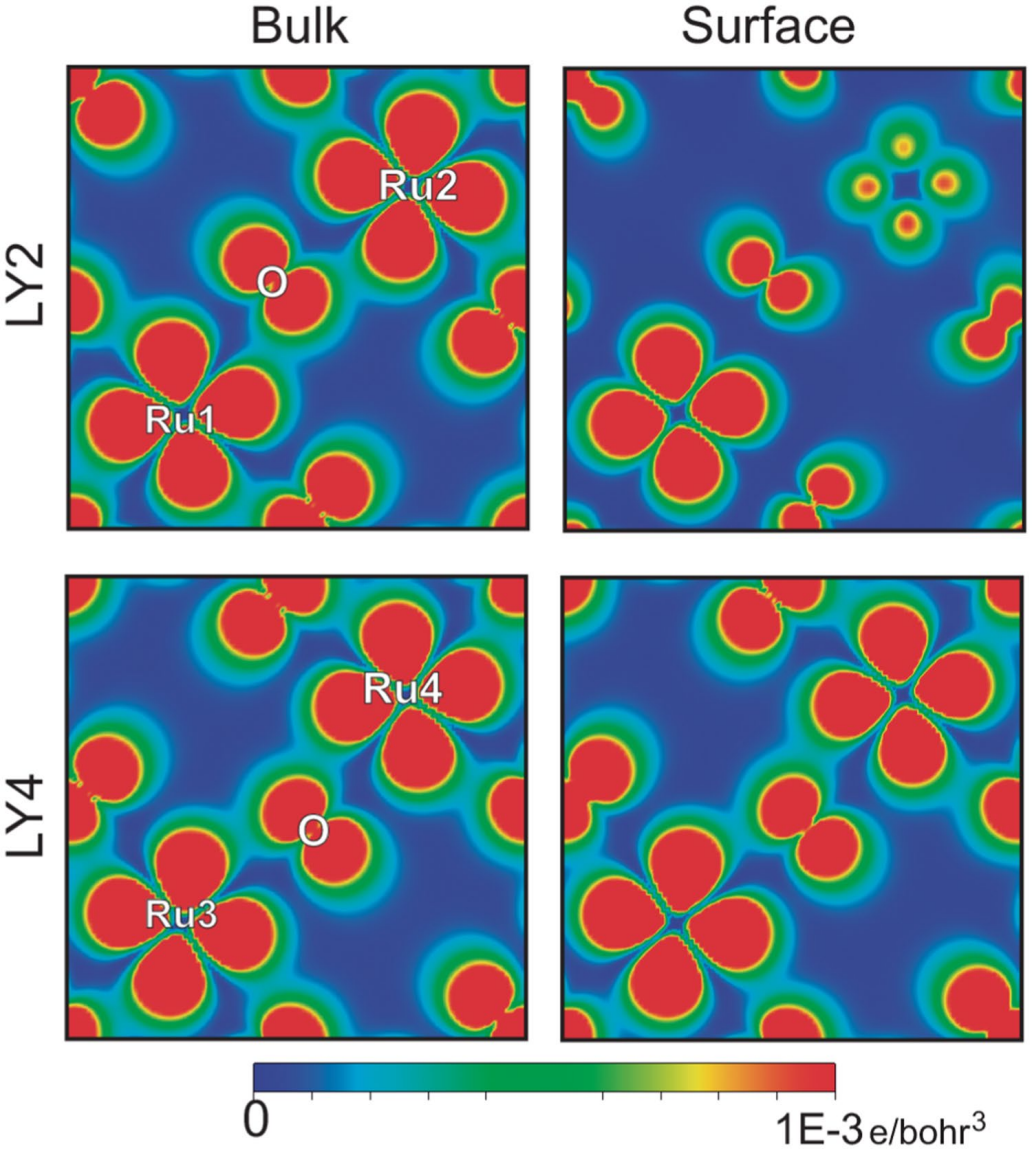
Calculated charge density of the LY2 and LY4 for bulk and surface structures for the range of ±0.1 eV around Fermi Energy.

These results show that surface symmetry breaking results from the creation of two distinct Ru environments characterized by different octahedral distortion, which causes difference in the charge density of these two sites that can clearly be seen in Fig. 2. The two different Ru environments will be referred to as Ru1O_6_ and Ru2O_6_. The Ru1O_6_ exhibits an in-plane compression and an out-of-plane elongation, resulting in a gain of charge of 0.055e and a magnetic moment equal to 1.44 *μ*_B_. In contrast, the Ru2O_6_ octahedron is stretched in-plane with an increase of the in-plane Ru2-O bond distance and a compressed out-of-plane. This leads to a loss of charge of 0.055e and a magnetic moment equal to 1.59 *μ*_B_ whereas, the bulk structure has only one type of Ru atom by symmetry with a magnetic moment equal to 1.41 *μ*_B_. It should be noted that the next RuO_2_ layer (LY4) shows smaller difference in Ru3-O/Ru4-O bond distances ($\simeq$0.005 Å) and, unlike LY2, it does not produce any appreciable changes in the electronic properties as compared to the bulk (see Fig. 2). These results clearly show that the symmetry breaking is purely localized.

**Figure 3:**
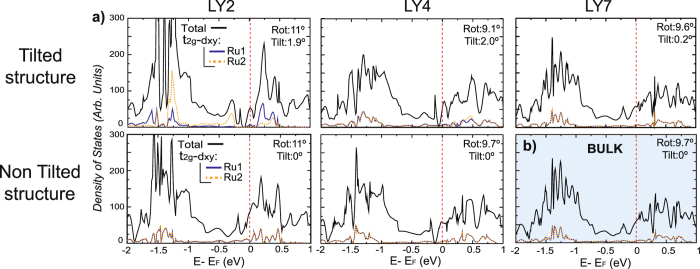
Total and projected onto Ru 4d-t_2g_-d_*xy*_ density of states (*α* + *β* contributions) for (**a**) surface RuO_2_ layers in the tilted and non-tilted structures and for (**b**) the bulk structure (no tilt). Rotation and tilt angles displayed in each plot.

To gain additional insight on the effects of symmetry breaking on the electronic properties at the surface, the density of states for tilted and untilted RuO_2_ layers along with the corresponding bulk density of states (DOS) are shown in Fig. 3. The SrO contributions around the Fermi energy (E_*F*_) are insignificant with the majority of electronic states coming from the RuO_2_ layers. As expected, bulk and non-tilted structures produce similar DOS. However, when the octahedra are tilted the total energy of the system is reduced by 11.6 meV per formula unit where a significant reduction of the states around E_*F*_ occurs. Specifically, the partial DOS yields different Ru1 and Ru2 4d_*xy*_ states at the surface due to symmetry breaking. The 4d_*xy*_ level of the Ru1 atom (compressive in-plane octahedra) becomes unoccupied due to a stronger coupling between O and Ru producing a larger splitting of the d_*xy*_ orbital, whereas the 4d_*xy*_ level in Ru2 is occupied. As a consequence there is a reduction of electronic states crossing E_*F*_, leading to a reduction in metallicity at the surface as compared to the bulk.

Non-tilted and tilted structures have similar octahedral rotation in the surface bilayer, which could indicate that rotation is not strongly coupled with electronic or magnetic properties. This result is in agreement with our previous simulations on bulk Sr_3_Ru_2_O_7_^1^, where we found that electronic and magnetic properties do not depend on the octahedral rotation. The difference in the electronic properties of both structures is due to tilt and therefore, octahedral tilt plays an important role in the observed properties of Sr_3_Ru_2_O_7_.

In our previous investigation on bulk Sr_3_Ru_2_O_7_^1^, we did not have the capability to treat the PM state. Thus, we found the FM state to always be the ground-state but it does become nearly degenerate with the AFM-I phase by −0.5 meV per formula unit. Here, we find that this competition disappears for the surface tilted structure where the FM state is favored with a total energy difference of 22 meV per formula unit.

**Figure 4:**
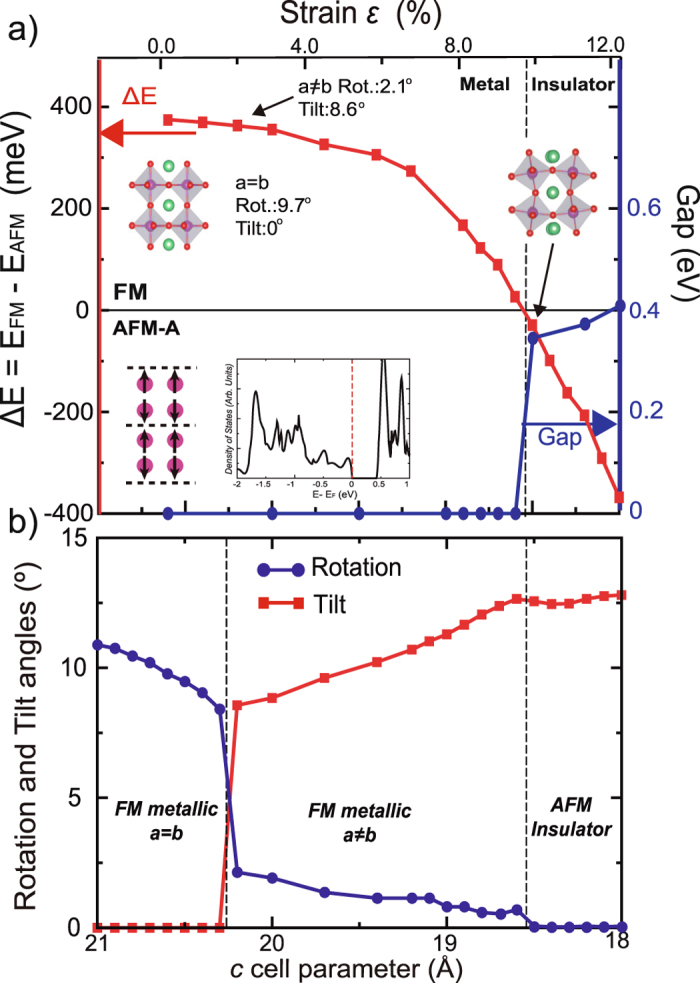
(**a**) Phase diagram of Sr_3_Ru_2_O_7_ showing the evolution of the ΔE and bandgap as a function of *c* cell parameter. The structural insets show the octahedra tilts in the ground-state structure and in the AFM-A insulator phase. The *α* + *β* density of states at *c* = 18.5 Å and AFM-A configuration are also displayed. (**b**) Evolution of RuO_6_ tilt and rotation as a function of *c* cell parameter.

Both experiment and theory show that RuO_6_ octahedral tilt reduces the metallicity of the system at the surface. The obvious extension of this finding is to calculate the effect on the bulk by the application of a uniaxial pressure along the *c* axis. Uniaxial pressure should induce tilt and change the delicate balance between FM-AFM spin states. We observe that the application of $\simeq$2% compressive strain (correspondent to $\simeq$1.5 GPa) produces a 8.6° octahedral tilt accompanied by a large reduction of rotation from 9.7° to 2.1° in the bulk. This point is marked by an arrow in Fig. 4a. However, the system still exhibits the FM ground state. By increasing the uniaxial pressure to $\simeq$21 GPa the system transforms to an AFM insulating ground state, marked by the second arrow in Fig. 4a.

Figure 4a displays the calculated bandgap as well as the difference in total energy between the FM and AFM-A states as a function of uniaxial pressure (along the *c* direction). For each uniaxial compressive strain a constrained geometry relaxation was performed where the atoms, *a* and *b* lattice parameters were allowed to relax while holding the *c* lattice parameter fixed. For high pressures the resulting AFM state is different from the lowest energy AFM-I phase found in the ground state bulk structure. This AFM insulating state obtained at 21 GPa, with *c* = 18.5 Å is an AFM-A state with a band gap of 0.35 eV. Further increasing uniaxial pressure does not significantly increase the band gap. Tilt of octahedra are $\simeq$12° similar to that found in the AFM insulator Ca_3_Ru_2_O_7_^14^. This provides additional evidence that the local structure is strongly coupled with spin and charge degrees of freedom. Finally, the mechanism associated with the AFM-A insulating phase transition can be understood via the inverse Goodenough-Kanamori interaction^20^. The application of uniaxial pressure forces the O atoms to move, resulting in the Ru-O-Ru intrabilayer angle (along the *c* axis) decreasing below 180° (i.e. tilt) along with a reduction of the corresponding Ru-O bond lengths. This produces a reduction of the magnetic coupling that satisfies the Goodenough-Kanamori rules^21, 22^ ultimately, leading to the FM-AFM phase transition.

Figure 4b shows the resulting tilt and rotation as the *c* axis reduces due to uniaxial pressure. It is clear that there are two first order phase transitions, one between the FM metallic (*a* = *b*) and the distorted FM metallic (a ≠ b) phases and the other, under higher uniaxial pressure, from the FM metallic to AFM insulator. Therefore, our calculations reveal two different hidden phase transitions in the bulk.

## Conclusion

By applying uniaxial pressure we have discovered a hidden AFM insulating phase in bulk of Sr_3_Ru_2_O_7_. The hypothesis of using uniaxial pressure to uncover this phase was motivated by our experimental and computational investigations on the structure property relationship due to: (1) The effects of RuO_6_ octahedra tilts on the electronic and magnetic properties of the first surface bilayer in Sr_3_Ru_2_O_7_ and (2) The strong FM-AFM competition found in the bulk phase. We observe that there is a strict relation between octahedral tilts and reduction of metallicity on the Sr_3_Ru_2_O_7_ surface as compared to the bulk. By applying $\simeq$21 GPa of uniaxial compressive strain along the *c* axis we predict a phase transition from FM metal to an AFM-A insulator whose AFM structure is different from the lowest energetically AFM ground-state structure (AFM-I). Furthermore, the mechanism that leads to the AFM-A state is based on the inverse Goodenough-Kanamori interaction and Goodenough-Kanamori rules.

## Methods

We have performed first principles DFT calculations based on PBES-10 hybrid functional. This functional is based on a mixing of 10% Hartree-Fock exchange with 90% PBESol exchange potential^23^, which has been shown to accurately capture the properties of Sr_3_Ru_2_O_7_^1^. For an additional explanation of this particular mixing the reader can be referred to ref. 24. We used the CRYSTAL14 computational package^25, 26^ which uses atom-centered Gaussian-type orbital (GTO) basis sets to build Bloch functions and thus expand the one-electron crystalline orbitals. The GTOs and calculation parameters used in this investigation have been reported in ref. 1. Surface Sr_3_Ru_2_O_7_ structures were modeled using slabs symmetrically terminated along the (001) direction (Fig. 1). The system under study has 25 layers with five RuO_6_ octahedral bilayers totaling 120 atoms. For atomic relaxation, the first 8 atomic layers, which includes two RuO_6_ bilayers were included in the relaxation process while the remaining atoms were fixed to the calculated bulk parameters^1^. To obtain a better understanding of the coupling between surface and octahedral distortion, and to determine the ground-state surface structure, simulations were performed with and without tilt. The total energy difference between tilted and untilted RuO_6_ octahedra is −11.6 meV per formula unit, indicating that having RuO_6_ tilted octahedra in the 1st surface bilayer is the preferred ground-state surface structure.

## Electronic supplementary material

Supplemental Material
