# Supplementary material for: Predicting hidden bulk phases from surface phases in bilayered Sr3Ru2O7
Source: Sci Rep. 2017 Aug 31;7:10265. doi: 10.1038/s41598-017-10780-6 (PMC5579013; doi:10.1038/s41598-017-10780-6)
Supplement: Supplementary file 1 — Supplemental Material [file 41598_2017_10780_MOESM1_ESM.pdf]

Predicting hidden bulk phases from surface phases in bilayered $Sr_3Ru_2O_7$

Pablo Rivero[1], Rongying jin[2], Chen Chen[2], Vincent Meunier[3], E. W. Plummer[2], and William Shelton[1,*]

[1]Center for Computation and Technology, Louisiana State University, Baton Rouge, Louisiana 70803, USA
[2]Department of Physics and Astronomy, Louisiana State University, Baton Rouge, Louisiana 70803, USA
[3]Department of Physics, Applied Physics, and Astronomy, Rensselaer Polytechnic Institute, Troy, NY 12180, USA
[*]wshelton@lsu.edu

ABSTRACT

The ability to predict hidden phases under extreme conditions is not only crucial to understanding and manipulating materials but it could also lead to insight into new phenomena and novel routes to synthesize new phases. This is especially true for Ruddlesden-Popper perovskite phases that possess interesting properties ranging from superconductivity and colossal magnetoresistance to photovoltaic and catalytic activities. In particular, the physical properties of the bilayer perovskite $Sr_3Ru_2O_7$ at the surface are intimately tied to the rotation and tilt of the RuO_6 octahedra. To take advantage of the extra degree of freedom associated with tilting we have performed first principles hybrid density functional simulations of uniaxial pressure applied along the c-axis of bulk $Sr_3Ru_2O_7$ where we find that the octahedra become tilted, leading to two phase transitions. One is a structural transition at $\simeq$ 1.5 GPa, and the other is from a ferromagnetic (FM) metal to an antiferromagnetic (AFM) insulator at $\simeq$ 21 GPa whose AFM spin configuration is different from the AFM state near the FM ground state.

Quantification of the uniaxial pressure

Here we analyze the stress tensor of $Sr_3Ru_2O_7$ as a function of the c lattice parameter to quantify the corresponding pressure applied along the [001] direction. In the ground-state structure the calculated diagonal components of this tensor (σ_{xx}, σ_{yy}, and σ_{zz}) are found to be less than 0.005 GPa while the off-diagonal terms (σ_{ij} i $\neq$ j) are less than 10^{-12} GPa. This provides a satisfactory estimation of the initial pressure conditions. The optimization of the atomic positions, and of the a and b lattice parameters as a function of decreasing c gives access to the stress tensors needed to quantify the uniaxial pressure applied on the [001] direction.

The diagonal components of the stress tensor are shown in Fig. 1 as a function of c cell parameter. As c is decreased, σ_{zz} increases while both σ_{xx} and σ_{yy} are kept nearly constant with corresponding pressures of less than 0.4 GPa for the entire range of c values. The value of the off-diagonal terms is found to be nearly zero for the same range of compressive strains. However, the evolution of σ_{zz} experiences two discontinuities as a consequence of the predicted phase transitions in $Sr_3Ru_2O_7$ induced by applying uniaxial pressure along the c axis.

We first observe a large reduction of about 3 GPa in σ_{zz} from 1.5GPa to -1.5GPa at c = 20.2 Å which corresponds to the transition from the non-tilted to the tilted structure. Once this transition is achieved, no uniaxial pressure applied along the [001] direction is needed to maintain the octahedral tilt in the structure and the system would stabilize in a metastable phase with $c \simeq 20$ Å. Further compressive strain in the [001] direction yields a linear increase of σ_{zz} while σ_{xx} and σ_{yy} remain nearly zero. At c = 18.5 Å we identify a second pressure release on σ_{zz} of about 4.5 GPa which corresponds to the FM metal to AFM insulating phase transition. After this transition, the linear response of σ_{zz} as a function of c parameter is recovered.

Given the relatively small stresses of the σ_{xx} and σ_{zz} and the negligible changes of the off-diagonal terms, we can estimate that an uniaxial pressure of around 1.5 GPa is needed to drive the system towards a tilted FM structure and around 21 GPa to the AFM insulator phase in $Sr_3Ru_2O_7$.

Ru(4d) orbital filling

The bulk $Sr_3Ru_2O_7$ structure has slightly elongated RuO_6 octahedra in its ground-state. As we apply uniaxial compressive strain along the c-axis, these octahedra become first quasi-regular and later compressed in the out-of-plane direction. These structural changes have important consequences in the orbital ordering and filling of the Ru($4d$) electrons in the compound that can affect the electronic and magnetic properties.

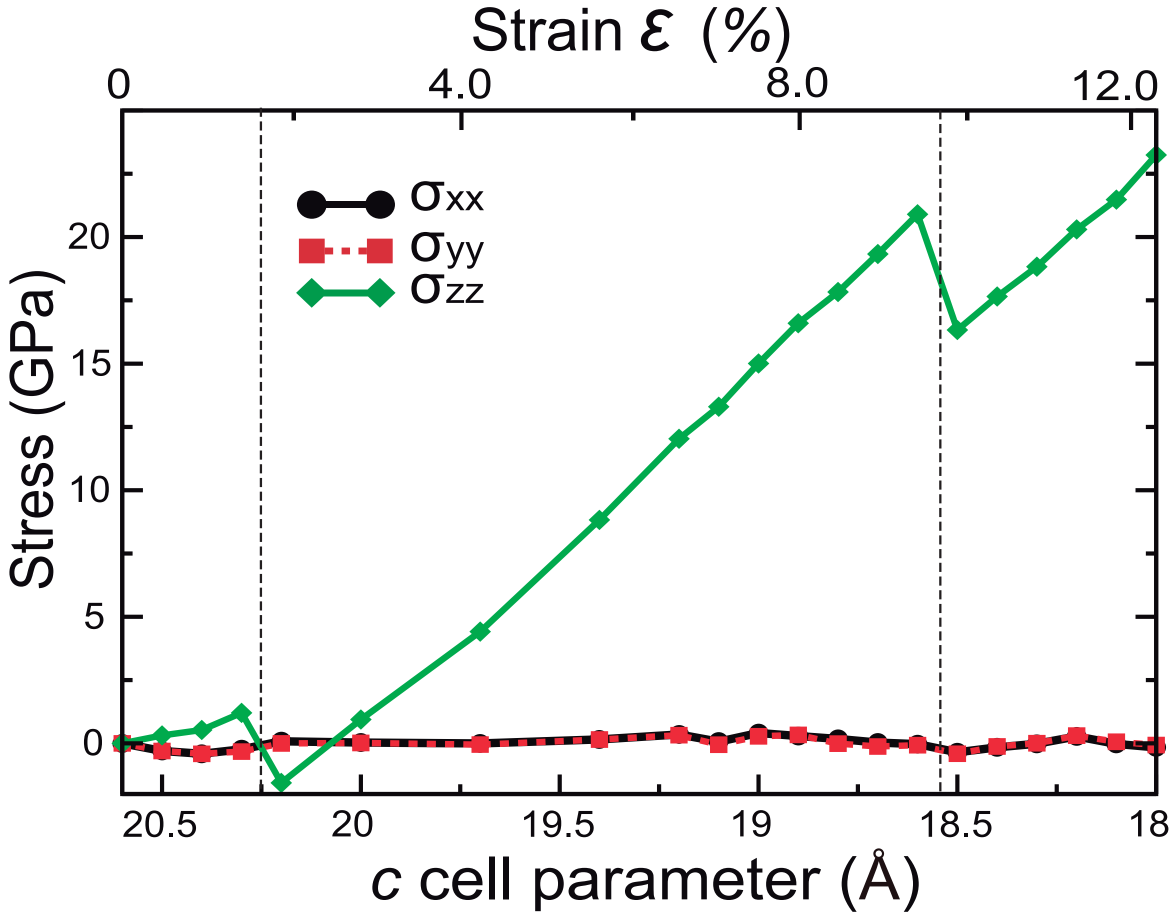

Figure 1. Evolution of the diagonal components of the stress tensor as a function of c cell parameter. Note, the off-diagonal terms remain zero for all the values of c considered.

In Fig. 2 we show the evolution of the 4d electron occupancies ($\alpha + \beta$) as a function of c lattice parameter. At c = 20.6 Å (0 GPa), xz and yz orbitals are doubly degenerated while xy forms a singlet at higher energy (relative to the doublet) and thus, it is less occupied. This t_{2g} symmetry breaking into a doublet and a singlet is a consequence of the elongated octahedra.

The first transition (structural) occurs at c = 20.2 Å and leads to a symmetry breaking of the xz and yz orbitals along a crossover where the xy orbitals are now at a lower energy and more occupied than the xz and yz orbitals. This is due to the structural change from elongated-to-compressed octahedra leading to a larger electron-electron repulsion with neighboring p-orbitals. This effect can also be seen in the z^2 and x^2-y^2 orbitals (although these orbitals are less populated than the t_{2g}).

As the uniaxial pressure increases (reduction of the c lattice parameter) the orbital fillings do not show appreciable changes until the metal-to-insulator and magnetic phase transitions occur at c = 18.5 Å ($\simeq$ 21 GPa). At this point, the energy splitting between xy and the xz and yz orbitals increases leading to a significant change in filling in favor of the xy orbitals. The compressed and tilted RuO_6 octahedra under this pressure makes the superexchange dominate the Ru-O-Ru out-of-plane interaction and the system stabilizes in the AFM-A insulating state.

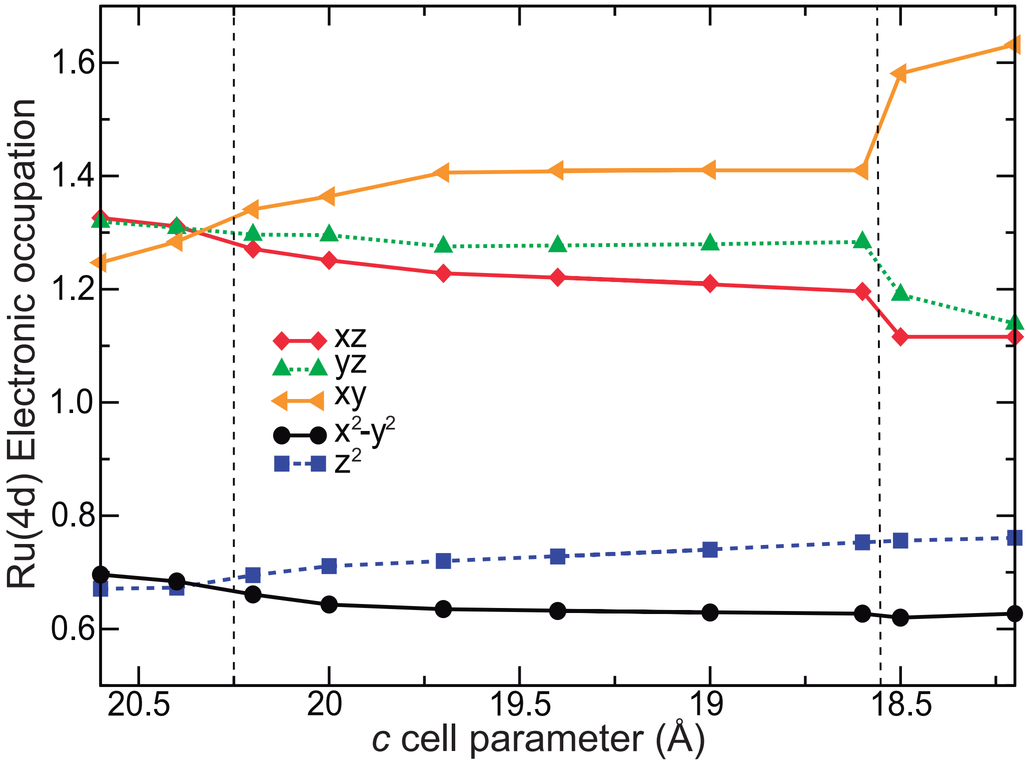

Figure 2. Evolution of the Ru(4d) electronic occupations ($\alpha + \beta$) as a function of c lattice parameter.
